# Therapeutic Effect of Glucose-Insulin-Potassium (GIK) Infusion Therapy in the Treatment of Acute Aluminum Phosphide Poisoning: An Institutional Study

**DOI:** 10.7759/cureus.76182

**Published:** 2024-12-22

**Authors:** Atta Ullah, Sakhi Jan, Hamid Shahzad, Maryam R Dar, Samrina Khan, Kashif Ahmad, Abdullah Abdullah, Muhammad R Khan, Maaz Obaid, Muhammad Khalid

**Affiliations:** 1 Emergency Medicine, Lady Reading Hospital, Peshawar, PAK; 2 Nephrology, Rehman Medical Institute, Peshawar, PAK; 3 Family Medicine, Khyber Medical University, Peshawar, PAK; 4 Neurology, Rehman Medical Institute, Peshawar, PAK; 5 Anesthesiology, Rehman Medical Institute, Peshawar, PAK; 6 Anesthesia Department, PGY2 Anesthesia, Lady Reading Hospital, Peshawar, PAK

**Keywords:** acute aluminium phosphide poisoning, acute poisoning, aluminium phosphide, aluminium phosphide poisoning, glucose insulin potassium infusion, pesticide causing cytochrome oxidase inhibition, pesticide poisoning

## Abstract

Background: Acute aluminum phosphide (ALP) poisoning presents a significant global medical challenge, particularly in regions where it is commonly used as a pesticide. Despite medical advancements, mortality rates from ALP poisoning remain high. Glucose-insulin-potassium (GIK) infusion therapy has emerged as a potential treatment for ALP poisoning due to its ability to counteract its toxic effects on metabolism and heart function. However, there is limited institutional data on the effectiveness of GIK therapy in treating ALP poisoning. Hence, this study aims to evaluate the therapeutic impact of GIK infusion therapy in managing acute ALP poisoning within our institution. The research aims to provide valuable insights into the efficacy of GIK therapy, with the potential to enhance patient outcomes and guide clinical practices in ALP poisoning management.

Materials and methods: This cross-sectional study was conducted at the Resuscitation Unit of the Emergency Department of Lady Reading Hospital (LRH), Peshawar, from February 1, 2021, to May 31, 2021, using convenient sampling of all patients with aluminum phosphide (ALP) poisoning meeting the inclusion criteria. The study group included 15 ALP poisoning cases treated with GIK infusion therapy combined with standard supportive measures and examined individuals with ALP poisoning admitted to the Resuscitation Unit during a specific period, comparing their outcomes to those observed before the introduction of GIK therapy. Randomization was based on time span, as GIK therapy was introduced in October 2020. Inclusion criteria encompassed a confirmed history of ALP poisoning with at least one deranged vital parameter, including patients of all genders and age groups. At the same time, we excluded patients with normal blood pressure on arrival, no deterioration four hours post-presentation, or those who expired before arrival. Data on hospital registration number, gender, age, arrival date, serial blood pressures, supportive measures, GIK infusion usage, and outcomes were analyzed using SPSS version 20.0 to assess the therapeutic impact of GIK infusion therapy.

Results: Among 15 ALP poisoning cases, 20% resulted in fatalities, with 80% either transferred to acute units (40%). Mortality was highest (66%) in the 20-29 age group, contrasting with no deaths in the 1-19 age group. Additionally, 33% mortality occurred in the 30-39 age group. Only 25% of patients with blood pressure below 91/61 succumbed. GIK therapy led to hemodynamic stability, prolonged unit stays, and increased transfers. Patients in acute units had longer stays compared to discharged or expired cases, reflecting the nuanced dynamics of ALP poisoning management.

Conclusion: This research aimed to systematically examine the effect of GIK infusion therapy on ALP poisoning cases at LRH, Peshawar, offering insights into its effectiveness compared to previous cases and reducing mortality by enhancing cardiac function.

## Introduction

Pesticides cause around 300,000 deaths annually worldwide. Aluminium phosphide is one of the most toxic substances, and it is used as a fumigant for stored cereal grains [1], insecticides, and rodenticides [2]. ALP is available in the form of tablets known as rice tablets [3], pellets, granules, or dust. Over the past 40 years, the growing use of aluminium phosphide (ALP) for both agricultural and non-agricultural activities has led to a rise in cases of poisoning attributed to ALP. Additionally, its widespread availability in developing nations has contributed to its increasing use in suicide attempts. It can easily be bought and has no effective antidote [4,5]. Commercially, dark grey ALP tablets of 3.0 g each consist of ALP (56%) and carbamate (44%) [6]. The lethal dose of aluminium phosphide is between 0.15 and 0.5 g (0.0053 and 0.0176 oz) [7].

On contact with gastrointestinal fluids, ingested formulations of ALP liberate phosphine gas, which is absorbed by gut mucosa. Phosphine not only partially inhibits cytochrome c oxidase but also interacts with hydrogen peroxide to form highly reactive hydroxyl radicals, which initiate lipid peroxidation. The combined effect causes a disturbance of mitochondrial morphology and leads to fatal complications such as gastritis, hepatic necrosis, disseminated intravascular coagulation, cardiac arrhythmia, metabolic acidosis, congestive heart failure, hypotensive shock, and eventual multiorgan failure [8].

An initial diagnosis of acute ALP poisoning is made based on history and/or clinical suspicion pertaining to typical signs and symptoms, which include cough, nausea, vomiting, diarrhoea, headache, fatigue, and dizziness. In severe exposure, lung irritation with persistent coughing, ataxia, paraesthesia, tremor, diplopia, hypotension, a weak pulse, and jaundice may also occur. A silver nitrate-impregnated paper test confirms the diagnosis by detecting the presence of phosphine/phosphide in the breath and gastric aspirates of exposed patients [9].

The management of acute ALP poisoning primarily involves serial monitoring, early resuscitation, and symptomatic treatment using supportive measures. Supportive treatment for ALP poisoning includes gastric lavage using oils, IV fluids, magnesium sulphate, vasopressors, sodium bicarbonate, steroids, and N-acetylcysteine. In addition, blood tests, including complete blood count, haemoglobin and haematocrit, arterial blood gases, coagulation profile, and renal and liver function tests, should be done to monitor organ systems. Chest X-rays and electrocardiography are also used to assess lung and heart function [10].

It predominantly affects women over men, with most cases occurring in adolescents and gradually decreasing in frequency among older age groups, with no instances reported in individuals aged 60 and above. Most cases manifest with blood pressure that cannot be recorded [11].

Although no specific antidote for ALP poisoning is available, glucose-insulin-potassium (GIK) infusion precipitating hyperinsulinemia-euglycemia, first introduced as a recommended treatment option in 2008, supposedly improves cell carbohydrate metabolism, increases both cardiac inotropy and systemic vascular resistance, and corrects acidosis by rectifying calcium antagonist or beta-blocker-induced hypoinsulinemia. As carbohydrates are preferable fuel substrates of the myocardium under stressful conditions, GIK infusion assists in enhanced uptake of carbohydrates and, therefore, results in improved cardiac function [12]. Glucose-insulin-potassium infusion therapy has long been advised in the additional management of ischaemia and reperfusion disturbances in cardiac patients. GIK infusion was first used in the 1960s for ischaemic heart diseases. GIK therapy has been reported to be beneficial in cardiac surgeries as a result of randomized controlled T-trials (RCTs); however, its clinical efficacy is still uncertain [13]. As regards its benefit in treating ALP poisoning patients, only a small number of researches conducted have shown positive results [12,14,15].

A recent study conducted in India consisting of 60 participants suffering from ALP poisoning showed that using GIK regime therapy along with treatment options resulted in a 26.6% decrease in the mortality of in-hospital cases compared to only supportive treatment. Although the GIK regime, along with supportive care, resulted in a longer duration of hospital stay for the patient, the ultimate outcome of the results proved beneficial [15].

Another similar study carried out resulted in a similar outcome, i.e., patients given GIK therapy for ALP poisoning spent a comparatively longer time at the hospital (60 hours in the GIK therapy group compared to 24 hours in the conservative treatment group). It also showed a comparatively lower mortality in patients given GIK therapy (50% compared to 72.7% in the conservative therapy group). After the initiation of the GIK regime, the mortality decreased by an overall 4.5% with each passing hour [14].

A study that compares the two treatment modalities while also highlighting the effectiveness and overall improvement in mortality rate with the use of the GIK regime along with conservative management has not yet been carried out in Pakistan.

As the mortality of ALP poisoning has been reported to be high, the need for a specific antidote and therapy is of utmost importance. The aim of this study is to correlate the therapeutic effect of GIK infusion therapy with improved cardiac function in ALP poisoning cases.

## Materials and methods

Study duration and design 

We conducted the study from February 1, 2021, to May 31, 2021. A cross-sectional study was conducted using a convenient sampling of all patients with ALP ingestion based on the inclusion criteria received at the Resuscitation Unit of the ED of LRH, Peshawar.

The ALP cases observed during the aforementioned months were considered in the study group (treated by GIK therapy + standard supportive measures).

Sample size

In the present study, considering the desired 95% confidence level, a sample size of 15 was calculated to observe the effect of GIK infusion therapy specifically on ALP poisoning cases.

Statistics

The results were compared to a total of 58 ALP cases (treated with standard supportive measures only) of the previously conducted study in the same department. Randomization was achieved by segregating cases and controls based on time span. As GIK Infusion Therapy was only initiated in October 2020 at the Resuscitation Unit of ED of LRH, Peshawar, during the four months of this study, ALP poisoning cases were treated using both GIK Therapy and standard supportive measures. The results from this study were compared to a study conducted previously throughout 2019, where GIK therapy was not used and all ALP poisoning cases were treated via standard supportive measures in the same department.

Inclusion criteria

The inclusion criteria were based on a history of ALP poisoning matching the description of the tablet taken by the patient as well as one of the vital parameters being deranged. Patients from both genders and all age groups were included.

Exclusion criteria

This was based on any patient having a blood pressure of more than 131/91 mmHg on arrival, no signs of deterioration four hours after presentation to the ER, or the patient expired before arrival at the emergency department.

ALP poisoning patient’s data, including hospital registration number, gender, age, date of arrival, serial blood pressures, supportive measures, use of GIK infusion, and patient’s final outcome, was noted and analyzed using the Social Package for Statistical Sciences version 20.0 (IBM Corp., Armonk, NY).

## Results

During the time period of this study, a total of 37 poisoning cases were received at the Resuscitation Unit of ED of LRH, Peshawar, out of which 15 satisfied the inclusion criteria of ALP poisoning (Table 1). Table 1 illustrates the distribution of patient ages included in this study, with 60% falling within the 20-29 age bracket. The patients considered in this study were treated using GIK therapy in addition to standard supportive measures. The data were analyzed and the results were compared to those of the prior study (treated only with standard supportive measures) conducted at the same department considering ALP poisoning patients of 2019.

**Table 1:** Patients age distribution (n=15)

Age	Frequency	Percent
1–19	5	33.3
20–29	9	60
30–39	1	6.7
Total	15	100

Our study showed that patients who presented with a blood pressure reading below 91/61 were a total of 12 patients, and mortality occurred in only three patients (25%), as shown in Table 2. Hence, there was a 69.7% relative reduction in risk in patients who were treated with the GIK regime along with conservative therapy. This study also showed an overall mortality of 20% in patients on the GIK regime along with conservative therapy compared to a previous study showing 73% mortality in patients not offered the GIK regime. Thus, it was shown that GIK therapy amounted to an almost 53% reduction in mortality. The only patient presenting with un-recordable blood pressure in the current study was discharged home with out-patient follow-up (Table 2).

**Table 2:** Impact of GIK therapy on mortality among patients with different initial blood pressure readings

Blood pressure on arrival	Patient outcome (On GIK + conservative regime)		
	Discharged home	Shifted to ward	Expired
Un- recordable	1	0	0
Below 61/41	0	0	0
62/42 to 91/61	4	4	3
92/62 to 131/91	1	2	0
Above 131/91	0	0	0

**Figure 1:**
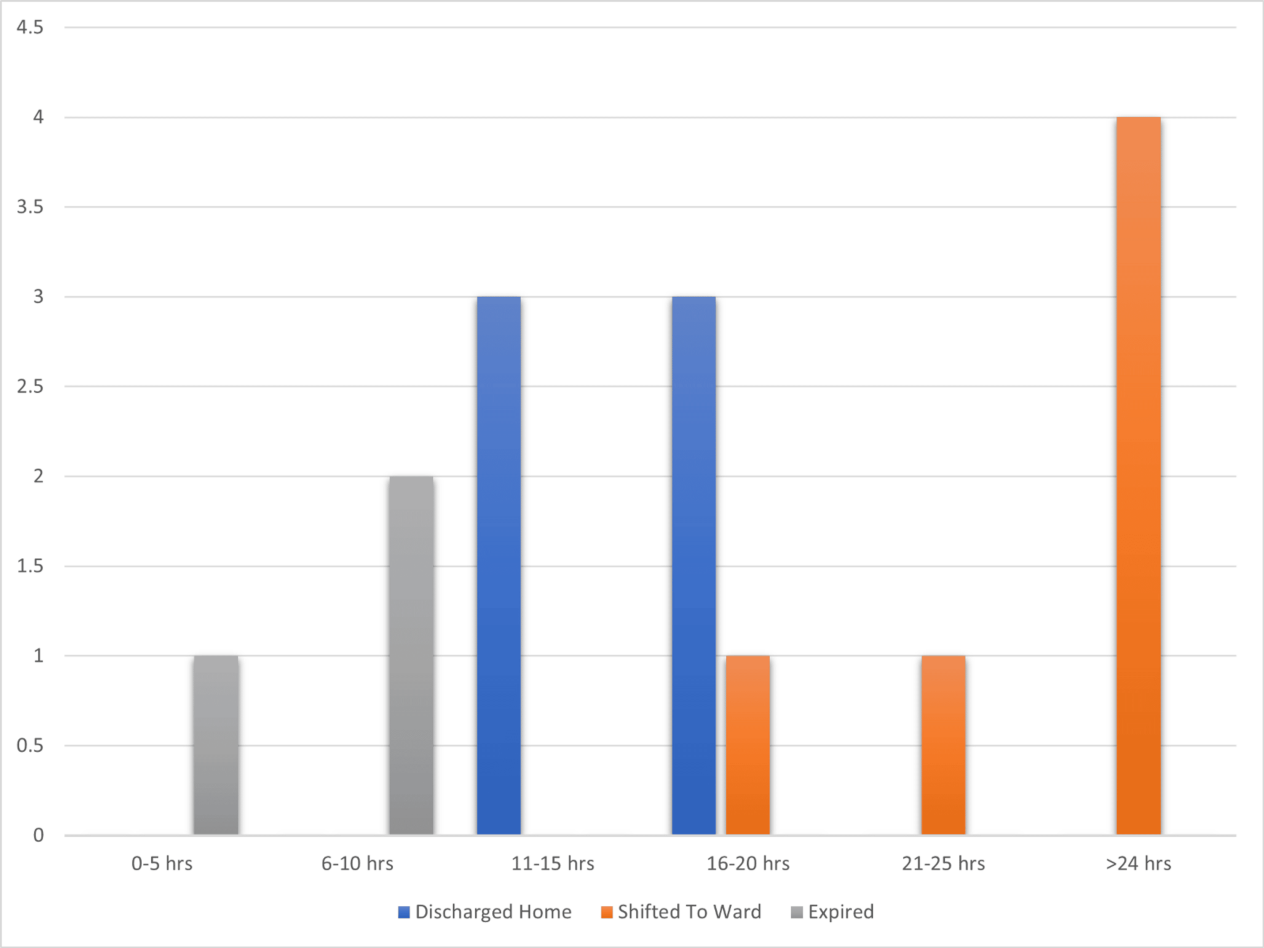
Length of hospital stay and patient outcome

Figure 1 shows that the majority of patients spent more than 10 hours in resuscitation. About 26.6% of patients were in each 16-20 hours group as well as those spending more than 24 hours.

**Figure 2:**
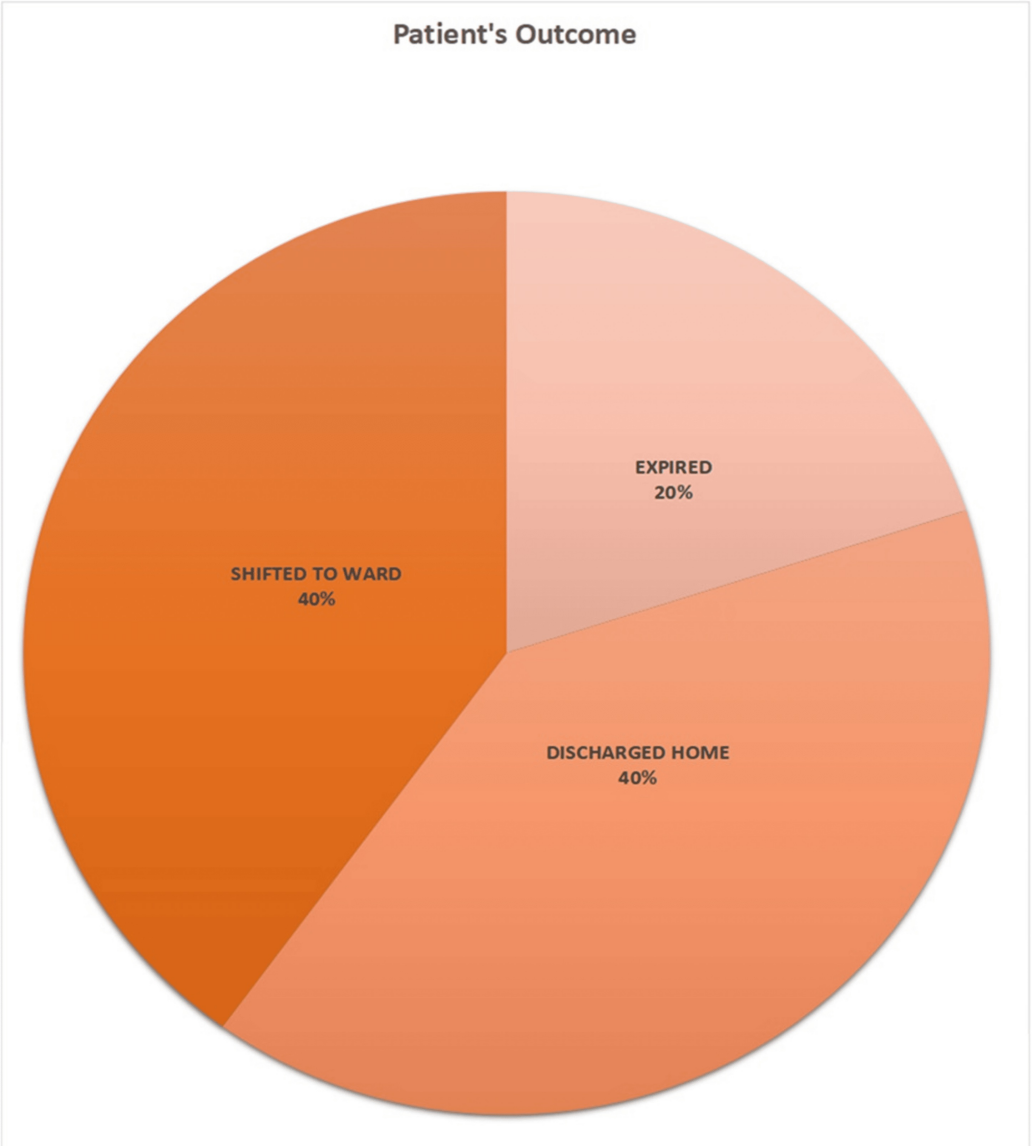
Outcome distribution of confirmed ALP poisoning cases: visualization of fatalities and patient dispositions

In the context of this study, out of a total of 15 confirmed cases of ALP poisoning, only 20% resulted in fatalities, while the remaining 80% were either transferred to an acute medical unit for further care (six patients) or discharged home (six patients), as depicted in Figure 2.

The distribution of patients by age group and their respective outcomes revealed that the largest proportion (60%) fell within the 20-29 age bracket. Notably, this age group exhibited a higher mortality rate (66% of total deaths) compared to the younger age group of 1-19 (zero deaths). Additionally, the age group of 30-39 reported a mortality rate of 33%, as detailed in Table 3.

**Table 3:** Age-wise distribution of patients and mortality rates in ALP poisoning cases: insights from the study

Patient age (years)	Discharged home	Shifted to ward	Expired
1–19	2	3	0
20–29	3	4	2
30–39	0	0	1
>40	0	0	0

Furthermore, from the records of serial blood pressure levels, it was observed that patients treated with levels and GIK therapy considerably improved or maintained their blood pressure with time. The most improvement (from the range 62/42 to 91/61 to the range 92/62 to 131/91, i.e., normal blood pressure) in blood pressure was reported in patients who were shifted to acute medical units for further management after initial resuscitation. On the other hand, the blood pressure of expired patients plummeted drastically while discharged patients maintained their blood pressure in the normal range (92/62 to 131/91).

The time each patient spent in the resuscitation unit was another intriguing observation. As GIK therapy resulted in haemodynamic stability, the length of stay in the unit was prolonged with regard to improvement in overall patient condition, and a rise in the number of patients shifted to acute medical units was noted. Patients shifted to acute medical units spent more time compared to discharged and expired patients, as evident in Figure 1.

## Discussion

In terms of improvement and overall survival of patients from GIK therapy in ALP poisoning, our study enhances the findings of previous such studies. Additionally, it demonstrates the understanding of practitioners in our locality regarding the use of this emerging regime to treat ALP poisoning.

Our study showed a drastic 54% reduction in mortality from ALP poisoning when patients were given the GIK regime along with standard conservative therapy. This finding is in accordance with another international study done by Pannu et al., which reported a 26.6% reduction in mortality when the regime was followed [15]. Although there is a difference in mortality reduction when comparing both studies, the difference can be, as a whole, attributed to multiple factors, including ethnicity, time of presentation of the patient to the ER, the overall health of the patient, and supportive care received. The overall improvement in mortality in both cases strongly supports the implementation of protocols that incorporate the GIK regime alongside conservative therapy in such cases.

As far as the hospital stay of a patient presenting with ALP poisoning is concerned, we can conclude from our study that putting the patient on GIK therapy prolongs the patient’s stay in the hospital. This can prove beneficial in terms of outcomes for the patient. Our patients showed that those who had stayed longer at the hospital, including both shifting patients to the ward or retaining the patient in resuscitation, showed decreased mortality compared to those with shorter time at the hospital. Those patients who had a shorter time at the hospital were based on not recovering from poisoning and eventually succumbing to it. Hassanian et al. reported similar results, doubling the time patients spent on the GIK regime compared to conservative therapy alone [12]. Mortality was also reportedly decreased by as much as 4% with each passing hour. Every 4 out of 5 patients in our study were either discharged home because of haemodynamic stability and no need for intervention or were shifted to a medical unit for further care.

In the previous study, which was done in the same hospital setting, it was observed that 42 out of a total of 58 ALP poisoning cases (confirmed by inclusion criteria) expired, which amounted to a mortality that approaches 73% [12]. In addition, it was noteworthy that among the 42 expired patients, all those who presented with blood pressure either unrecordable or below 91/61 died, while only two patients survived in the blood pressure range between 62/42 and 91/61. In short, the mortality of patients presenting with compromised blood pressure was shown to be very high (94.7%). Compared to this, the current study showed that patients who presented with blood pressure readings below 91/61 were a total of 12 patients, and mortality occurred in only three patients (25%). Hence, there was a 69.7% relative reduction in risk in patients who were treated with the GIK regime along with conservative therapy. This study also showed an overall mortality of 20% in patients on the GIK regime along with conservative therapy compared to a previous study showing 73% mortality in patients not offered the GIK regime. Thus, it was shown that GIK therapy amounted to an almost 53% reduction in mortality (73% vs. 20%).

Comparing with the previous study in the same setting, which showed the age-wise distribution of mortality in each age group as follows: 39.66% in the age group 1-19, 17.24% in the age group 20-29, 8.62% in the age group 30-39, and 6.89% mortality in patients more than the age of 40 years [12]. The current study showed different statistics demonstrating 66% of total mortality in this age group, 20-29, compared to no mortality in the younger age group of 1-19. Along with this, 33% of mortality was reported in the age group 30-39. This can conclude that the GIK regime has improved results along with conservative therapy in patients in the age group 1-19.

## Conclusions

Our statistical analysis demonstrates that the incorporation of GIK therapy, when combined with standard supportive treatment, enhances the overall survival rates of patients suffering from ALP poisoning. Consequently, our findings advocate for the utilization of GIK therapy alongside supportive measures in the management of ALP poisoning cases. Additionally, our study reveals that while GIK therapy may extend the duration of hospitalization for patients, it is associated with a concurrent improvement in survival outcomes.
